# Global trends in the application of nanopore sequencing technology in the detection of infectious disease pathogens: a bibliometric analysis from 2014 to 2024

**DOI:** 10.3389/fmed.2025.1610063

**Published:** 2025-07-23

**Authors:** Jiali Long, Benhua Zeng, Jia Li, Juan Zhang, Guohong Deng

**Affiliations:** ^1^Department of Infectious Diseases, Southwest Hospital, Third Military Medical University (Army Medical University), Chongqing, China; ^2^Chongqing Key Laboratory of Viral Infectious Diseases, Chongqing, China

**Keywords:** nanopore sequencing, pathogenic microorganisms, bibliometric analysis, real-time, genomic surveillance, antimicrobial resistance

## Abstract

**Purpose:**

This study aimed to comprehensively analyze the global landscape, trends, and research focus of nanopore sequencing technology in the field of pathogenic microorganism diagnosis using bibliometric analysis.

**Methods:**

Literature published between January 2014, and December 2024, was retrieved from the Web of Science Core Collection. A cross-sectional bibliometric analysis was conducted using VOSviewer, CiteSpace, Origin 2024, and R software to extract and evaluate metrics. Publications were categorized by country, institution, author, journal, highly cited papers, and keywords. Variables were compared based on publication output and academic impact, which included citation counts, citation impact, H-index, journal impact factor, total link strength, major pathogens, and research directions.

**Results:**

Initial searches identified 2,098 articles related to nanopore sequencing and pathogenic microorganisms, of which 729 were ultimately included in the analysis. Among the 104 participating countries, the United Kingdom, the United States, and China have led in publication output, citations, and academic influence. The most versatile institution was the University of Oxford, followed by Zhejiang University. The most productive scholars and journals were Crook, Derrick W., and Frontiers in Microbiology, respectively. Keyword analysis revealed that the primary advantages of nanopore sequencing include portability, long-read capabilities, and real-time analysis. Current research hotspots focus on real-time pathogen identification, viral genomic surveillance, and antimicrobial resistance profiling.

**Conclusion:**

Presently, nanopore sequencing is rapidly transitioning from laboratory research to on-site sequencing and public health emergency scenarios. To our knowledge, this study is the first bibliometric analysis to comprehensively delineate the latest developments in nanopore sequencing in pathogenic microorganism diagnosis. It provides researchers with an understanding of the current situation, identifies knowledge gaps, and points out future research directions.

## 1 Introduction

The rapid and accurate identification of pathogenic microorganisms is critical for clinical diagnosis, outbreak surveillance, and antimicrobial stewardship (1–3). Conventional culture-based methods and polymerase chain reaction (PCR)-dependent assays, while foundational in microbiology, face limitations in turnaround time, resolution, and scalability, particularly when confronting polymicrobial infections, unculturable pathogens, or emerging strains with novel genetic signatures (4). Recently, the advent of third-generation sequencing technologies, such as nanopore sequencing, has revolutionized pathogen detection by enabling real-time, high-throughput, and portable genomic analyses (3, 5).

Nanopore sequencing is characterized by its unique ability to generate ultra-long reads (> 100 kb) and directly detect epigenetic modifications, circumventing the amplification biases inherent in short-read sequencing platforms (6). These features are critical for addressing the complex genomic architectures of pathogens, including antibiotic resistance gene clusters, virulence plasmids, and repetitive elements, which are often fragmented or misassembled by traditional sequencing approaches (7–9). Furthermore, its portability and minimal infrastructure requirements make it a transformative tool for on-site diagnostics in resource-limited settings. This assertion is evidenced by its successful utilization in real-time monitoring of viral transmission during outbreaks of Ebola virus (EBOV) (10), Zika virus (ZIKV) (11), and severe acute respiratory syndrome coronavirus 2 (SARS-CoV-2) (12). Recent studies have demonstrated the utility of nanopore sequencing in a variety of clinical scenarios, including the identification of pathogens in bloodstream infections within 6 h, real-time metagenomic analysis of cerebrospinal fluid, and rapid detection of multidrug-resistant tuberculosis (13–15).

Despite these advances, there is a paucity of comprehensive reviews examining previous publications, academic influence, and research trends in the application of nanopore sequencing technology to pathogenic microorganisms across the globe. Bibliometric analysis has been extensively employed to explore the productivity of countries, institutions, and researchers in specific disciplines, identify key events, and predict emerging trends. The application of bibliometric analysis, which quantifies the distribution patterns and characteristics of scientific data from multiple perspectives, has the potential to reveal the overarching knowledge of architecture and the evolutionary trajectory of this field. Such insights empower experts and newcomers to delineate the scope of their discipline, discover novel topics of interest, and strategically formulate future research plans.

## 2 Materials and methods

### 2.1 Data source and search strategy

**Figure 1:**
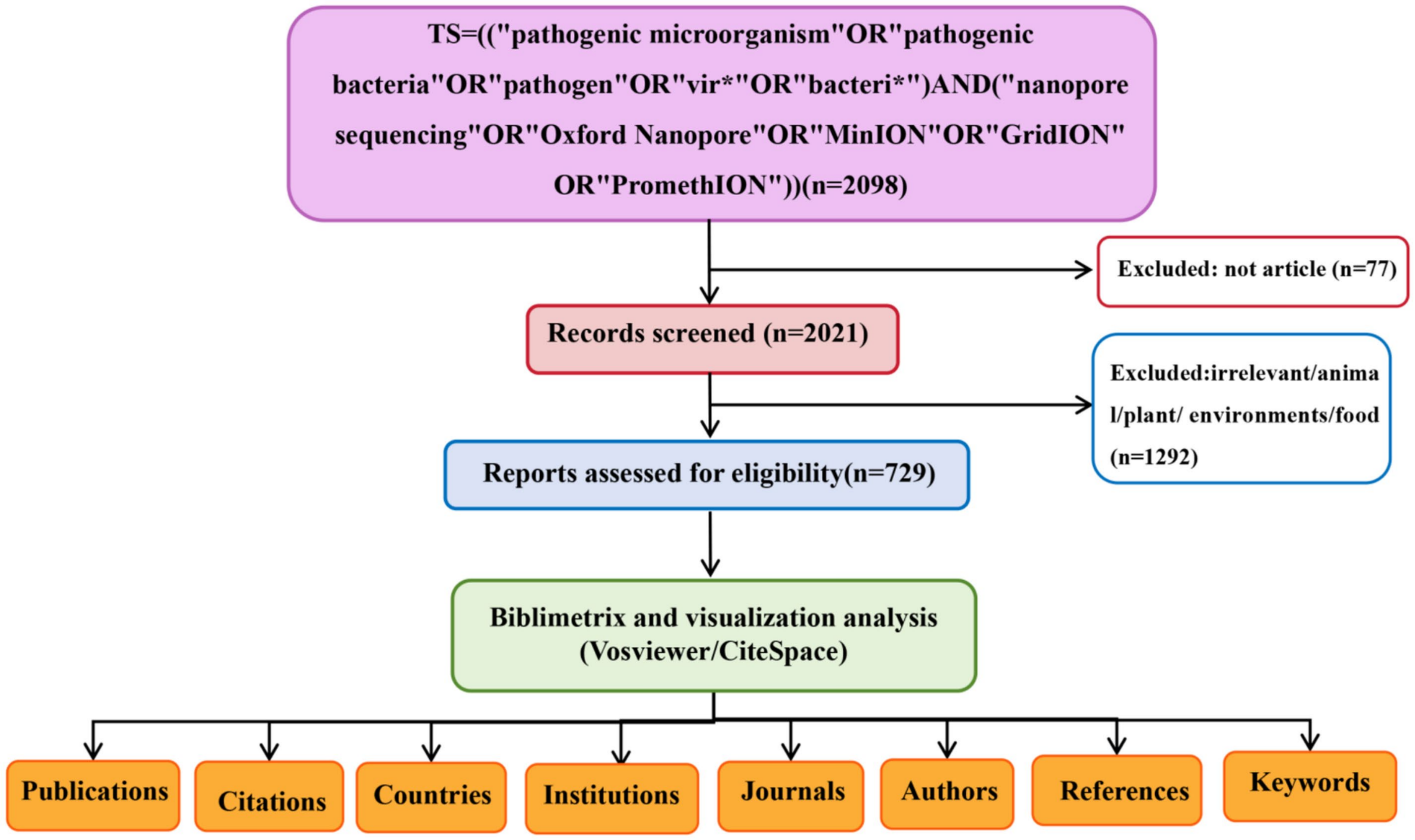
Flowchart of metrology screening.

We retrieved the Web of Science Core Collection (WOSCC) database to identify publications on nanopore sequencing from 2014 to 2024. To comprehensively capture literature relevant to the research topic while minimizing the inclusion of non-relevant records, we used the topical search query: TS = ((“pathogenic microorganism” OR “pathogenic bacteria” OR “pathogen” OR “vir*” OR “bacteri*”) AND (“nanopore sequencing” OR “Oxford Nanopore” OR “MinION” OR “GridION” OR “PromethION”)). Language was limited to English. A literature search and data download was performed on May 25, 2025. The screening process is summarized in Figure 1.

### 2.2 Bibliometric analysis

The VOSviewer software (version 1.6.20) was used to cluster countries/regions, institutions, journals, and keywords. The CiteSpace software (version 6.3.1) was used to visualize the institutional centrality cooperation authors’ network, generate keyword timeline maps, and conduct burst word analysis. Origin2024 software was used for statistical analyses. R software (version 4.4.1) was used to perform the geographical distribution of nanopore sequencing in the field of pathogenic microorganisms. Academic influence is a primary criterion for evaluating the academic excellence of the document, journal, institution, researcher, etc., and its measurement is inherent and diverse. We used the composite metrics to measure academic influence, including the following elements: (1) citation count: number of times a country/institution/author/journal is cited; (2) citation impact: average number of citations a document received; (3) Hirsh index (H-index): a researcher has an h-index if they have at least h publications for which they received at least h citations; (4) total link strength: total strength of the co-authorship links of a given country/institution/author with others; (5) impact factor (IF): functional approximation of the mean citation rate per citable item (16).

## 3 Results

### 3.1 Baseline characteristics of the eligible documents

**Figure 2:**
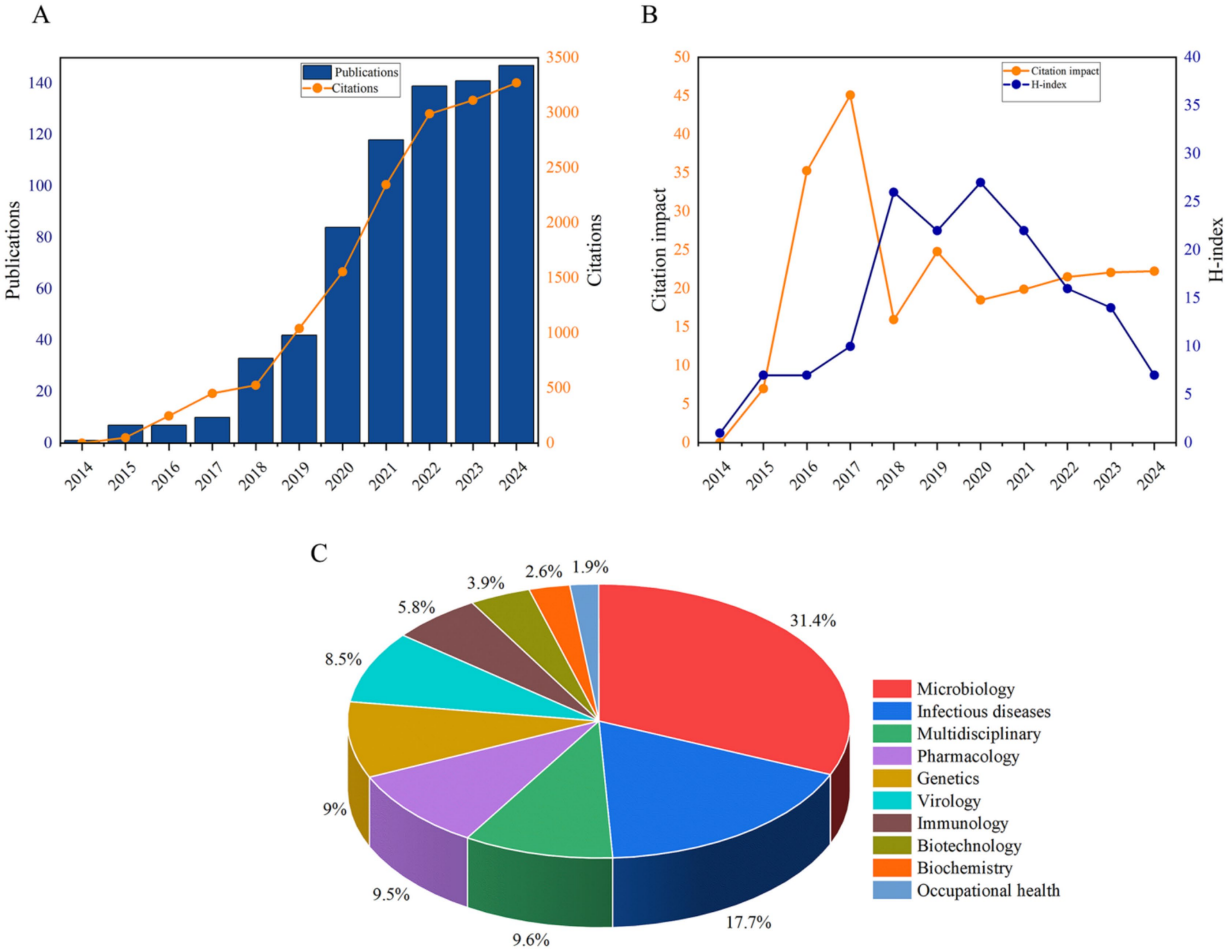
Baseline bibliometric characteristics of all eligible documents. **(A)** Annual publications and citations. **(B)** Citation impact per year and H-index. **(C)** Research areas.

The initial search identified 2,098 publications (Figure 1). After excluding non-article types (*n* = 77), 2,021 articles underwent rigorous title and abstract screenings. Studies unrelated to human pathogens or focusing exclusively on animal, plant, environmental, or food-related contexts (*n* = 1,292) were excluded, resulting in 729 eligible studies for final inclusion. The annual publication volume and citation counts from 2014 to 2024 are presented in Figure 2A. A consistent growth trend was observed for both metrics, with a notable surge post-2019. Before 2019, fewer than 35 publications annually addressed nanopore sequencing in pathogenic microorganisms. However, annual output has exhibited sustained growth since 2019, peaking at 147 publications in 2024. Citation counts followed a similar trajectory, reaching a maximum of 3,271 in 2024. Compared to 2015, citation impact demonstrated a modest increase, with significant upward shifts observed in 2016 and 2017 (Figure 2B). The 2020 year demonstrated unparalleled scholarly impact, attaining the maximum H-index of 27 (Figure 2B). Regarding the research areas, approximately 1/3 of the papers (*n* = 287, 31.4%) belonged to the microbiology category, followed by infectious diseases (*n* = 162, 17.7%), multidisciplinary (*n* = 88, 9.6%), pharmacology (*n* = 87, 9.5%), and genetics (*n* = 82, 9%) (Figure 2C).

### 3.2 Analysis of countries and institutions

**Figure 3:**
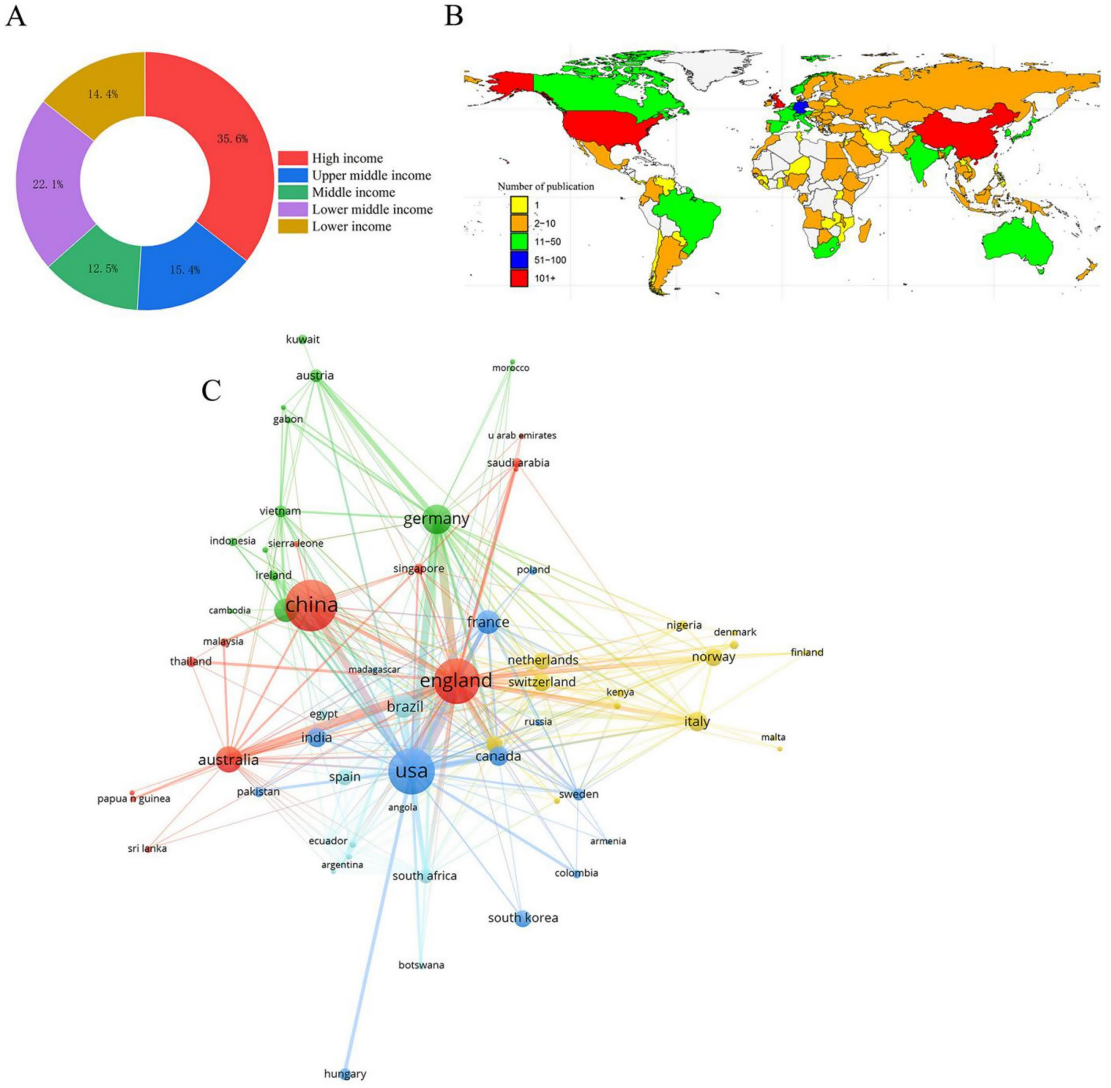
Relationships and clusters of countries. **(A)** Classification of countries according to income levels. **(B)** Geographical distribution based on the number of documents. **(C)** Relationship between international and domestic collaborations.

One hundred and four countries contributed to the global discourse, of which 35.6% were high-income countries, 15.4% were upper-middle-income countries, 12.5% were middle-income countries, 22.1% were lower-middle-income countries, and 14.4% were lower-income countries (Figure 3A). The global productivity map revealed pronounced geographical clustering, with most publications originating from Asia, North America, and Europe (Figure 3B). China, the USA, and England have been identified as the leading contributors to nanopore sequencing research because of their high productivity levels, citations, and H-index (Figure 3C). Subsequently, we analyzed the number of documents, citations, H-index, and main research pathogens of the top 10 most productive countries (Table 1).

**Table 1:** Top 10 most productive countries regarding nanopore sequencing and pathogen research from 2014 to 2024.

Country	Documents	Citations	Citation impact	H-index	Total link strength	Main pathogen
China	174	2,294	13	24	38	SARS-CoV-2, *K. pneumoniae*, *E. coli, M. tuberculosis*, HPV
USA	142	5,803	41	30	155	SARS-CoV-2, HSV, *K. pneumoniae*, *E. coli*, HBV
England	136	7,437	55	35	177	SARS-CoV-2, *K. pneumoniae*, DENV, IAV, *E. coli*
Germany	59	3,292	56	17	79	SARS-CoV-2, MPXV, *S. aureus*, EBOV, RVA
Australia	47	1902	40	18	51	*K. pneumoniae*, DENV, SARS-CoV-2, *P. aeruginosa,* HBV
France	39	1,537	39	14	49	DENV, SARS-CoV-2, *K. pneumoniae*, HBV, *P. aeruginosa,*
Japan	39	750	19	14	29	HCV, *K. pneumoniae*, HIV, SARS-CoV-2, *E. coli*
Brazil	37	1,034	28	10	67	DENV, SARS-CoV-2, ZIKV, CHIKV, *K. pneumoniae*
Canada	26	3,171	122	11	51	SARS-CoV-2, EBOV, *K. pneumoniae*, HBV, *M. tuberculosis*
India	26	253	10	8	13	SARS-CoV-2, *K. pneumoniae*, *Brucella, E. coli, P. aeruginosa*

**Figure 4:**
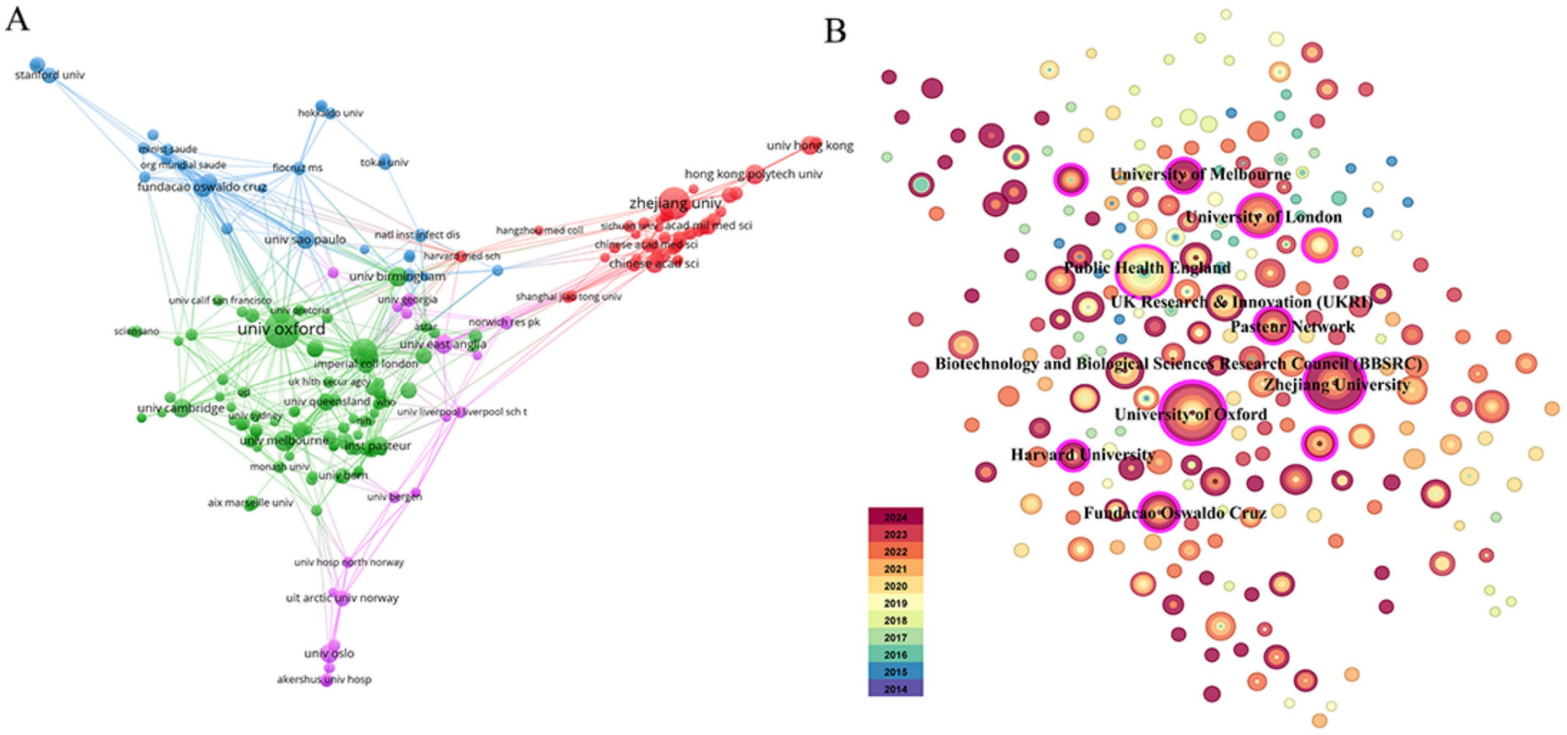
Visualization of institutions. **(A)** Collaboration network of institutions. **(B)** Top 10 institutions for related publications.

A total of 281 institutions participated in this collaboration. Figure 4A displays the institutional co-authorship network, comprising 211 institutions and 691 collaborative instances, with the University of Oxford and Zhejiang University serving as representative institutions in their respective countries. Additionally, these two organizations maintain close collaboration with domestic and international academic institutions. Figure 4B and Table 2 present the top 10 institutions with the highest documents in the fields of nanopore sequencing technology and pathogenic microorganisms.

**Table 2:** Top 10 productive institutions regarding nanopore sequencing and pathogen research from 2014 to 2024.

Institution	Documents	Citations	Citation impact	H-index	Main research
University of Oxford	43	2027	47	19	Epidemiology (SARS-CoV-2, DENV); genomic surveillance (ZIKV, DENV, influenza, HCV, CHIKV, HMPV); antibiotic resistance (*E. coli*, *K. pneumoniae, M. tuberculosis*)
Zhejiang University	35	422	12	10	Rapid diagnosis (bloodstream infections, bacterial pneumonia); antibiotic resistance (*K. pneumoniae*, *P. aeruginosa*, *E. coli*, *S. aureus, A. baumannii*)
Public Health England	25	4,093	164	19	Epidemiology (Lassa fever, influenza); rapid diagnosis (LRI); genomic surveillance (EBOV, ZIKV, SARS-CoV-2, CHIKV, dengue virus); antibiotic resistance (*S. aureus*, *M. tuberculosis*, *K. pneumoniae*)
University of London	22	1899	86	13	Epidemiology (DENV, Lassa fever, *M. tuberculosis*); genomic monitoring (EBOV, influenza, *S. pneumoniae*, *K. pneumoniae*); antibiotic resistance (*K. pneumoniae*, sexually transmitted)
Fundacao Oswaldo Cruz	19	930	49	8	Genomic surveillance (DENV, ZIKV, SARS-CoV-2, CHIKV); antibiotic resistance (*P. aeruginosa*)
UK Research & Innovation	18	930	52	12	Rapid diagnosis (LRI); Rapid identification (FMD virus); Epidemiology (SARS-CoV-2); antibiotic resistance (HBV, *Shigella* species)
Biotechnology and Biological Sciences Research Council	18	922	51	12	Rapid identification (FMD virus); Epidemiology (SARS-CoV-2); antibiotic resistance (*Shigella* species, *Neisseria gonorrhoeae, E. coli*)
Pasteur Network	15	1,155	72	9	Rapid diagnosis (*M. tuberculosis*); genomic surveillance (SARS-CoV-2; DENV, HBV); antibiotic resistance (*K. pneumoniae*, *P. aeruginosa*, *A. baumannii*)
University of Melbourne	15	1,085	72	10	Genomic monitoring (SARS-CoV-2, *S. pneumoniae*, RSV); antibiotic resistance (*K. pneumoniae*, *A. baumannii*)
Harvard university	13	1,076	83	7	Epidemiology (*Candida auris*); genomic surveillance (SARS-CoV-2, HBV, CHIKV); antibiotic resistance (*K. pneumoniae*)

### 3.3 Analysis of authors, journals, and highly cited publications

**Figure 5:**
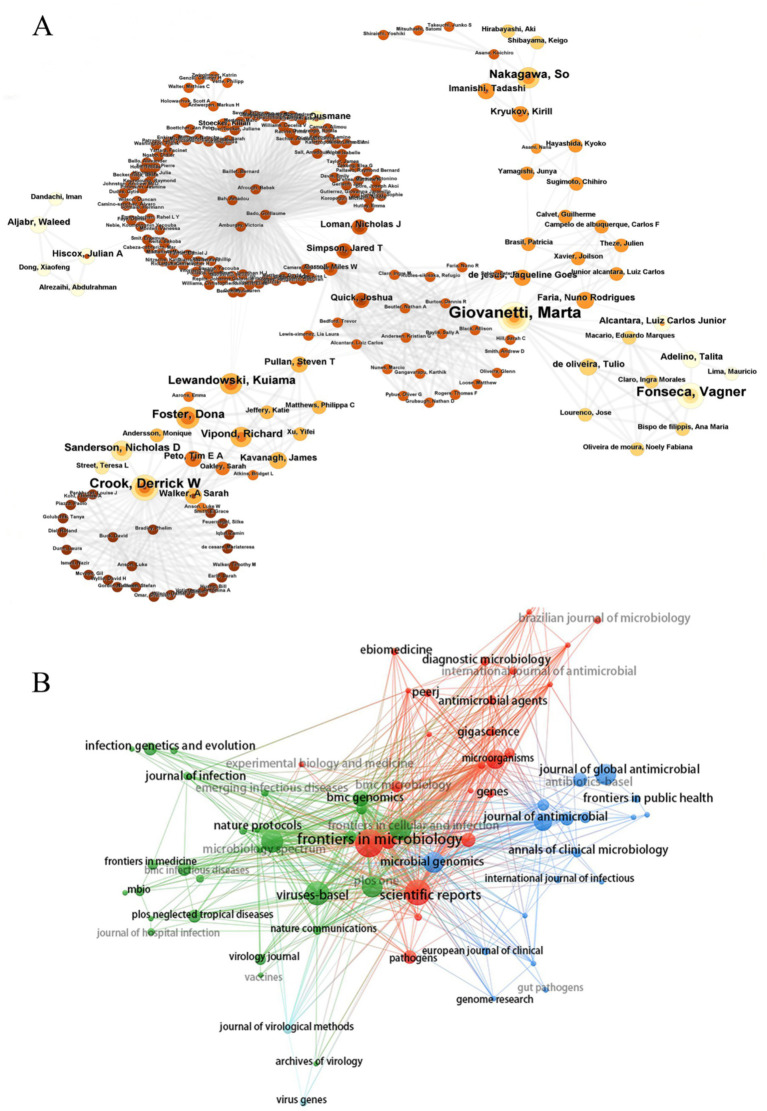
Visualization of authors and journals **(A,B)**.

Among the 5,393 authors included, the most prolific authors in the bibliometric analysis were Crook, Derrick W (*n* = 14), followed by Giovanetti, Marta (*n* = 12), Alcantara, Luiz Carlos Júnior (*n* = 12) (Table 3). Giovanetti, Marta and Alcantara, Luiz Carlos Júnior had the highest citation impact; both worked at the Fundacao Oswaldo Cruz. The centralized author clustering revealed a strong cooperative relationship between the authors (Figure 5A). The top three academic journals out of 205 were Frontiers in Microbiology (*n* = 43; Table 4), Scientific Reports (*n* = 35), and Viruses-Basel (*n* = 31). The journal with the highest impact factor was the Frontiers in Cellular and Infection Microbiology (IF = 4.6), followed by the Microorganisms (IF = 4.1). These journals were categorized into four clusters based on their co-citation relationships (Figure 5B).

**Table 3:** Top 10 productive authors of nanopore sequencing and pathogen research from 2014 to 2024.

Author	Institution	Documents	Citations	Citation impact	H-index
Crook, Derrick W	University of Oxford	14	844	60	11
Giovanetti, Marta	Fundacao Oswaldo Cruz	12	882	74	7
Alcantara, Luiz Carlos Júnior	Fundacao Oswaldo Cruz	12	882	74	7
Sanderson, Nicholas D.	University of Oxford	10	379	38	9
Fonseca, Vagner	Universidade do Estado da Bahia	9	101	11	5
Xavier, Joilson	Universidade Federal de Minas Gerais	9	157	17	7
Song, Hongbin	PLA, Ctr Dis Control & Prevent	9	96	11	6
Boldogkoi, Zsolt	University of Szeged	8	162	20	5
de Filippis, Ana Maria Bispo	Fundacao Oswaldo Cruz	8	114	14	5
Ruan, Zhi	Zhejiang University	8	77	10	5

**Table 4:** Top journals ranked by publication counts.

Journal	Documents	Citations	Total link strength	IF
Frontiers in Microbiology	43	637	185	4
Scientific Reports	35	798	159	3.8
Viruses-Basel	31	191	84	3.8
Frontiers in Cellular and Infection Microbiology	26	193	81	4.6
Microbiology Spectrum	26	153	66	3.7
Microbial Genomics	24	1,005	81	4
Journal of Global Antimicrobial Resistance	24	137	7	3.7
Plos One	23	315	85	2.9
Microorganisms	20	258	61	4.1
Infection and Drug Resistance	20	109	8	2.9

Table 5 displays the top 10 original articles that received the most attention in the application of nanopore sequencing in the field of pathogenic microorganisms. All HCPs were published before 2021, consistent with the expected trend that older articles inherently accumulate higher citation counts over time compared to recent ones. A study titled “Real-time, portable genome sequencing for Ebola surveillance,” published in “Nature” in 2016, garnered 967 citations, rendering it the most highly cited publication in this domain. In contrast, an analysis of HCPs from the past 3 years indicated that research efforts have increasingly concentrated on integrating targeted amplification with nanopore sequencing. This synergistic approach optimizes sequencing workflows by reducing costs, accelerating turnaround time, and enhancing accuracy (Table 6).

**Table 5:** Top 10 cited original articles.

Title	Institution	Journal	Year	Citation	IF
Real-time, portable genome sequencing for Ebola surveillance	University of Birmingham	Nature	2016	967	50.5
A complete bacterial genome assembled de novo using only nanopore sequencing data	University of Birmingham	Nature methods	2015	823	36.1
Multiplex PCR method for MinION and Illumina sequencing of Zika and other virus genomes directly from clinical samples	University of Birmingham	Nature protocols	2017	724	13.1
Completing bacterial genome assemblies with multiplex MinION sequencing	University of Melbourne	Microbial Genomics	2017	634	4
Rapid pathogen detection by metagenomic next-generation sequencing of infected body fluids	University of California	Nature medicine	2021	418	58.7
Nanopore metagenomics enables rapid clinical diagnosis of bacterial lower respiratory infection	University of East Anglia	Nature biotechnology	2019	406	33.1
Rapid antibiotic-resistance predictions from genome sequence data for *Staphylococcus aureus* and *Mycobacterium tuberculosis*	University of Oxford	Nature communications	2015	374	14.7
Rapid metagenomic identification of viral pathogens in clinical samples by real-time nanopore sequencing analysis	University of California	Genome medicine	2015	362	10.4
MinION nanopore sequencing identifies the position and structure of a bacterial antibiotic resistance island	Public Health England	Nature biotechnology	2015	319	33.1
Identification of bacterial pathogens and antimicrobial resistance directly from clinical urines by nanopore-based metagenomic sequencing	University of East Anglia	Journal of antimicrobial chemotherapy	2017	215	3.9

**Table 6:** Top 10 original articles that have been cited the most times in the last 3 years.

Title	Institution	Journal	Year	Citation	IF
Rapid spread of SARS-CoV-2 Omicron subvariant BA.2 in a single-source community outbreak	University of Hong Kong	Clinical Infectious Diseases	2022	61	8.2
Comparison of R9.4.1/Kit10 and R10/Kit12 Oxford Nanopore flowcells and chemistries in bacterial genome reconstruction	University of Oxford	Microbial Genomics	2023	55	4
Nanopore sequencing of a monkeypox virus strain isolated from a pustular lesion in the Central African Republic	Inst Pasteur	Scientific Reports	2022	44	3.8
Metagenomic prediction of antimicrobial resistance in critically ill patients with lower respiratory tract infections	University of California	Genome Medicine	2022	40	10.4
Nanopore is preferable over Illumina for 16S amplicon sequencing of the gut microbiota when species-level taxonomic classification, accurate estimation of richness, or focus on rare taxa is required	The BioArte Limited	Microorganisms	2023	36	4.1
Genomic epidemiology insights on NDM-producing pathogens revealed the pivotal role of plasmids on *bla*_NDM_ transmission	Zhengzhou University	Microbiology Spectrum	2022	36	3.7
Analysis of global *Aeromonas veronii* genomes provides novel information on source of infection and virulence in human gastrointestinal diseases	University of New South Wales	BMC Genomics	2022	33	3.5
Oxford nanopore long-read sequencing enables the generation of complete bacterial and plasmid genomes without short-read sequencing	Chinese Center for Disease Control & Prevention	Frontiers in Microbiology	2023	28	4
Direct detection of drug-resistant *Mycobacterium tuberculosis* using targeted next generation sequencing	New York State Department of Health	Frontiers in Public Health	2023	27	3
Rapid detection of bacterial pathogens and antimicrobial resistance genes in clinical urine samples with urinary tract infection by metagenomic nanopore sequencing	Yantai University	Frontiers in Microbiology	2022	27	4

### 3.4 Keywords of research hotspots

**Figure 6:**
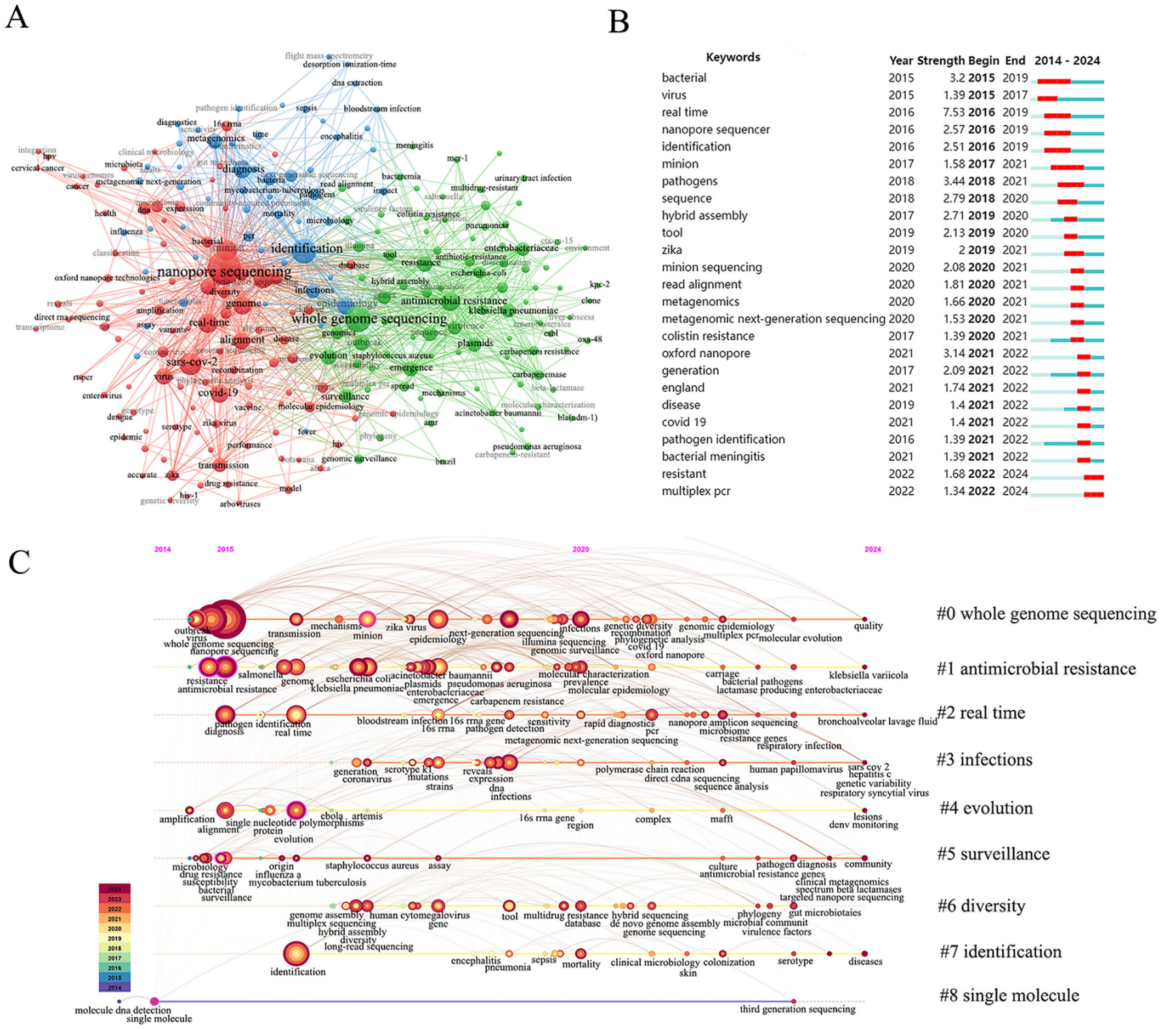
Keyword analysis. **(A)** Cluster visualization map of keywords analysis. **(B)** Top 25 keywords with the strongest citation bursts generated. **(C)** Keyword timeline clustering map.

Keywords are of paramount importance in reflecting core themes research interests and future directions in a given discipline. As illustrated in Figure 6A the keywords have been divided into three clusters. The first major cluster displayed in blue focuses on the application of nanopore sequencing in bacterial identification and diagnosis with keywords such as identification diagnosis metagenomics epidemiology and bloodstream infection. The second major cluster is in red and emphasizes the real-time surveillance capabilities of nanopore sequencing with keywords such as real-time SARS-CoV-2 alignment genome and virus. The third major cluster displayed in green highlights the applications of nanopore sequencing in the context of antimicrobial resistance (AMR) with keywords including whole genome sequencing antimicrobial resistance *Klebsiella pneumoniae* (*K. pneumoniae*) plasmids and *Enterobacteriaceae.*

Most significantly, the terms “resistant” and “multiplex PCR” (2022–2024) exhibited citation burst times that persisted into 2024, indicating sustained recognition and interest in these areas (Figure 6B). Notably, “real-time” (2016–2019, burst strength = 7.53) and “pathogens” (2018–2021, burst strength = 3.44) emerged as the strongest burst terms, reflecting heightened attention to real-time pathogen identification by nanopore sequencing in public health and research during these periods. The keyword timeline revealed a dynamic evolution of nanopore sequencing technology (Figure 6C). Early development and validation (2014–2016) were mainly focused on the functional exploration of nanopore sequencing in the identification and diagnosis of pathogenic microorganisms. The key terms included nanopore sequencing, whole genome sequencing, antibiotic resistance, identification and real-time. Technical optimization and clinical transformation (2017–2021) were widely used in the identification and drug resistance analysis of pathogens such as *K. pneumoniae*, *Pseudomonas aeruginosa* (*P. aeruginosa*), and *Enterobacter,* and molecular epidemiological analysis of ZIKV and other viruses by whole genome sequencing. Rapid development and diversification (2022–2024) have been deeply applied to real-time screening of drug resistance genes, targeted capture of pathogens, and multi-omics analysis of complex samples.

## 4 Discussion

### 4.1 General information

Nanopore sequencing is a revolutionary technology that enables real-time analysis of DNA or RNA by detecting transient changes in the ionic current as nucleic acids traverse engineered protein nanopores (17). Unlike conventional sequencing methods that rely on amplification or fluorescent labeling, this single-molecule approach eliminates PCR-induced biases. It directly identifies epigenetic modifications (5-methylcytosine and N6-methyladenosine), thereby distinguishing itself from short-read sequencing platforms that depend on synthetic probes (18). The defining advantages of this technology, namely ultra-long read lengths, on-site deployable portability, and cost-effectiveness, have propelled its application in identifying pathogenic microorganisms, analysis of antibiotic resistance, and epidemic monitoring (19).

The current bibliometric analysis delineates the status and trends of nanopore sequencing technology in the field of pathogenic microorganisms. The studies were stratified into two distinct phases based on whether the number of publications exceeded 50 per year for two consecutive years. From 2014 to 2019, nanopore sequencing-related research was in a slow growth stage. Since 2019, it has entered a rapid growth stage, with more than 50 publications annually. Notably, a competitive landscape has emerged between China, which contributes the largest number of publications (174 documents), and England, which obtains the most citations (7,437 citations). Among the 10 institutions with the highest productivity, five are in England. These findings highlight the important contributions and leading position of England in nanopore sequencing application research. This leadership may be attributed to the country’s economic situation, high level of investment in healthcare, and the presence of industry leader Oxford Nanopore Technology (ONT) in the country. Since its establishment in 2005, ONT has been committed to the development of DNA sequencing technology based on nanopores and launched the first commercial nanopore sequencer, MinION, in 2014, which has brought revolutionary progress to sequencing technology. Professor Crook, Derrick W, from Oxford University, has published fourteen publications and is the author with the most prolific in the field. Since 2015, Professor Crook, Derrick W has explored the wide application of nanopore sequencing in the field of pathogenic microbiology, including the identification of influenza virus (20), *Neisseria gonorrhoeae* (21), human metapneumovirus (22), and antibiotic resistance analysis (23, 24). HCPs typically represent fundamental themes in a research field. In this study, the top 10 most-cited publications primarily focused on the rapid diagnosis of pathogens, epidemic surveillance, and antibiotic resistance analysis, aligning with the identified research hotspots. Further exploration of the differential applications of nanopore sequencing in these three key research areas across various pathogens could offer enhanced guidance for clinical practice and scientific investigations.

### 4.2 Current research status and development trends

#### 4.2.1 Nanopore sequencing in rapid identification of pathogens

The rapid identification of pathogens is vital for shortening diagnostic timelines, guiding targeted antimicrobial therapy, reducing healthcare costs, and minimizing unnecessary hospitalizations, thereby having significant implications for clinical management and public health systems. Compared to conventional diagnostic technologies (for example, culture-based methods and PCR), nanopore sequencing exhibits distinct advantages in the following aspects. Bias-free and ultra-broad detection capability: Without preset targets, it can simultaneously identify bacteria, fungi, viruses, and parasites, particularly advantageous for screening rare or emerging pathogens. For example, identifying pathogens such as *R. typhi* (25), *A. multifidum* (26), *Tropheryma whipplei* (27), *Mycobacterium marinum* (28), and *Streptococcus suis* (29) and other pathogens that lack typical symptoms and are easily missed. Real-time detection capability, output results during sequencing, and the quality and status of samples can be understood early in sequencing. Currently, pathogen identification and culture-based antimicrobial susceptibility testing (AST) may take 3 days or longer. Direct sequencing and predictive AST on the positive blood culture broth of 201 patients with suspected sepsis. Results the species-level consistency between AST predicted by nanopore sequencing and conventional species identification methods was 94.2%, which increased to 100% in single microbial infection. The potential turnaround time from labeled positive blood culture to report generation could be achieved within 9–17 h, which was faster than conventional AST methods (up to 48 h or longer). This result indicates that nanopore sequencing can reduce the generation cycle of clinical reports while ensuring diagnostic performance (7). Charalampous et al. (1) developed a nanopore metagenomics protocol for diagnosing bacterial lower respiratory infections (LRI) and conducted experimental tests on respiratory samples from 40 suspected patients with LRI. The results revealed that the sensitivity and specificity were 96.6 and 41.7%, respectively, which were better than those of conventional culture, and the pathogen identification and antibiotic resistance genes could be completed within 6 h. Technological innovation capability, recent analysis of HCPs have revealed a growing body of research focusing on the integration of targeted amplification with nanopore sequencing (Table 6). Nanopore targeted sequencing (NTS) has been developed using amplicon sequencing principles for the rapid detection of hundreds of pathogens and has exhibited significant advantages in pathogen detection in respiratory tract infections (30), bloodstream infections (13), and central nervous system infections (14). In bloodstream infection diagnostics, NTS achieved higher positivity rates (69.5% versus 33.9%) and accuracy (85.3% versus 53.2%) than blood cultures, with 50% of results generated within 223.5 min and 95% within 419 min (13). Moreover, it has a low-biomass pathogen detection capability, which can detect pathogens at ultralow concentrations (< 10 copies/μL) in challenging specimens, such as cerebrospinal fluid. These characteristics are uniquely valuable in diagnosing and treating acute and severe diseases, including sepsis and central nervous system infections (31).

#### 4.2.2 Nanopore sequencing in epidemic surveillance and public health emergency response

Nanopore sequencing technology has emerged as a critical tool for epidemic surveillance and public health emergency response due to its real-time capabilities, portability, and broad-spectrum detection capacity, demonstrating unique value in identifying emerging pathogens and tracking viral mutations. In 2014, the ONT launched the first MinION sequencer, a device whose instrument size is only the size of a U disk and whose weight is less than 100 grams. This device can be connected to desktops or laptops for detection and real-time analysis, making it well-suited for use in settings with limited resources, such as refugee camps and remote clinics, or for on-site testing during outbreaks. As early as 2015, Quick et al. (10) transported the MinION sequencing system to Guinea via standard airline luggage for EBOV genome sequencing. Results were generated within 24 h of receiving Ebola-positive samples, with sequencing taking only 15–60 min. By integrating sequencing data from Guinea and Sierra Leone, this study identified frequent cross-border transmission between the two regions. This pioneering work demonstrates the potential for rapid deployment of the MinION system for outbreak monitoring, thereby shifting epidemic response strategies from “passive detection” to “active genomic surveillance.” In 2016, Brazil’s ZIKV real-time analysis project established a mobile laboratory equipped with MinION systems for amplicon-targeted sequencing, which increased the scale of virus genome monitoring and promoted ZIKV sequencing in various parts of Brazil. Phylogenetic analysis of the virus genome revealed the origins of closely related genotypes, which is vital for studying the evolutionary laws of pathogens, outbreak tracing, and epidemic prevention and control (11). Notwithstanding the aforementioned advantages, nanopore sequencing faces considerable challenges in the context of large-scale genomic initiatives. When ONT data was the only source used, the assemblies contained a high number of indels and frameshifts, resulting in misassemblies and erroneous gene annotations (32). In the prediction of AMR and the phylogenetic analysis of *Neisseria gonorrhoeae* isolates, ONT data produced phylogenetic tree topologies that were comparable to those produced by Illumina datasets, and all related isolates were clustered similarly. However, the number of isolates was limited and the genomic heterogeneity of the strains was high, which may have exerted a slight bias on the analysis (33). Currently, there are few reports on the on-site nanopore sequencing in sudden outbreak zones, likely due to the unpredictability, dangerousness, and control orientation of such scenarios. Beyond the monitoring of viral genomes through on-site nanopore sequencing, this method has also been employed in a series of critical research studies in the laboratory. The simultaneous sequencing of numerous samples and the real-time surveillance of the viral genome have facilitated the establishment of epidemiological links during periods of sustained viral transmission. In addition to the previously mentioned examples of EBOV and ZIKV, related studies have been conducted on Chikungunya virus (34), dengue virus (35), influenza A virus (36), yellow fever virus (37), Lassa fever virus (38), and SARS-CoV-2 (12). Concurrently, nanopore sequencing has played a pivotal role in bacterial outbreak investigations, including those involving wild-type *Klebsiella* var*iicola* (39)*, Clostridioides difficile* (40), *Salmonella* (41), *Enterococcus faecium* (42), *K. pneumoniae* (43). These studies integrate genomic and epidemiological data to facilitate public health laboratories in comprehending and monitoring the patterns and diversity of bacterial/viral epidemics, expeditiously tracking and investigating infections in hospital and community settings, and ascertaining opportunities for infection control interventions to further mitigate healthcare-associated infections.

Notably, early sequencing devices exhibited constrained output capabilities, rendering them inadequate for large-scale research initiatives. This limitation was ultimately addressed by innovative advancements in flow cell design, notably with the introduction of the PromethION platform by ONT. The platform can achieve a theoretical throughput of 290 Gb per flow cell, and an impressive 14 Tb for the PromethION 48 (3). However, the cost of nanopore sequencing for DNA methylation studies in Alzheimer’s disease and frontotemporal dementia is $1,000 per sample, representing a 4-fold increase compared to Illumina-based approaches (44). In order to enhance competitiveness, ONT must further reduce costs, thereby improving accessibility and cost-effectiveness across diverse applications.

#### 4.2.3 Nanopore sequencing in AMR detection

AMR has become a major threat to global health. From 1990 to 2021, more than 1 million people died annually from drug-resistant infections. It is estimated that by 2050, AMR will directly cause more than 1.9 million deaths per year and indirectly cause more than 8 million deaths (45). AMR infections are primarily driven by pathogens, including ESKAPE (*Enterococcus faecium*, *Staphylococcus aureus*, *K. pneumoniae*, *Acinetobacter baumannii*, *P. aeruginosa*, and *Enterobacter*) or *Mycobacteria*, and form antibiotic resistance genes (ARGs) through horizontal gene transfer between microbial communities. Rapid, comprehensive, and accurate detection of ARGs is crucial for guiding early and effective antibacterial treatment (46). Compared to culture-based susceptibility testing (2–5 days), nanopore sequencing, with its long reads and real-time data generation capabilities, can rapidly construct a complete bacterial genome in a matter of hours and align it to a known antibiotic resistance gene database to identify ARGs, thereby guiding clinical targeted drugs in a timely manner. Currently, The nanopore sequencing method has been demonstrated to be effective in the detection of drug resistance in various pathogens, including *K. pneumoniae* (47), *P. aeruginosa* (48), *Salmonella* (49), *Mycobacterium tuberculosis* (50), *Neisseria gonorrhoeae* (51, 52). This has resulted in significant advancements and accomplishments in the realm of detecting ARGs, identifying single nucleotide mutations and heterogeneous drug resistance, and analyzing drug resistance mechanisms. For instance, Liu et al. (53) utilized nanopore sequencing to identify three novel *K. pneumoniae* sequence types (STs) from 1,484 clinical isolates carrying the mobile multidrug-resistant (MDR) efflux pump gene cluster (*tmexCD1-toprJ1*), including a pan-resistant ST22 strain co-harboring *bla*_KPC-2_ and *bla*_NDM-1_ (carbapenemase genes), a MDR ST3691 strain, and a MDR ST37 strain. The identification of these novel strains represents a valuable addition to the epidemiological study of *K. pneumoniae* carrying *tmexCD-toprJ*, highlighting the ability of nanopore sequencing in detecting AMR caused by horizontal transmission of drug-resistant genes facilitated by mobile elements, such as plasmids. Subsequent to this, the same research team utilized nanopore sequencing to ascertain that the tandem repeats and copy number of the *bla*_SHV-12_ gene element of cefiderocol-resistant strains had increased. This finding suggests the potential for a novel mechanism contributing to cefodil resistance in *K. pneumoniae*, and the hypothesis that was validated through quantitative PCR and *in vitro* experimentation. In a separate study, Sauerborn et al. (7) demonstrated the great potential of real-time genomics for rapid and accurate analysis of complex bacterial infections in clinical settings through the case of a multidrug-resistant *K. pneumoniae* infection. In comparison with a clinically established diagnosis, real-time genomics-based drug resistance prediction can identify a novel antibiotic resistance gene variant, *bla*_KPC-14_, located on the low-abundance IncN plasmid. This finding is of great significance for clinical decision-making and potential patient prognosis, indicating the potential for change by integrating real-time genomic analysis into clinical practice.

Two significant advancements in nanopore sequencing, namely Q20 + chemistry and adaptive sampling, have had a substantial impact on real-time pathogen identification, viral genome surveillance and AMR analysis. The implementation of the latest Q20 + chemistry and flow cell has been demonstrated to enhance the precision of base detection data and the accuracy of sequencing. In comparison with the old kit LSK109 with R9.4, the average sequence accuracy of SARS-CoV-2 sequencing was increased from 96.25 to 98.34% using the Q20 + kit (LSK112) with flow cell R10.4, and the proportion of sequences with an accuracy of more than 99% was increased from 0.61 to 30.1% (54). Furthermore, three strains of *Salmonella* carrying MDR plasmids were sequenced using the LSK114 kit with flow cell R10.4.1, and *de novo* genome assembly was performed with accuracy increased from 92 to 98% and Q20 increased from 13 to 42% (55). Nanopore adaptive sampling (NAS) improves the target reading by real-time alignment, and ejects the uninteresting read by reversing the voltage across the nanopore, which is very important for the targeted enrichment of low-abundance genomes. Herbert J. et al. conducted a study to assess the impact of the nanopore sequencing chemistry version on the output of the novel adaptive sampling approach. In comparison with non-adaptive sequencing, this resulted in a 5-7-fold increase in target enrichment (LSK109: 4.8-fold; LSK112: 6.8-fold) (56). It is noteworthy that no significant differences in adaptive sampling enrichment efficiency were observed between the older and newer ONT sequencing chemistries, suggesting that adaptive sampling performs consistently across different library preparation kits. Hang Cheng et al. developed a metagenomic workflow that is based on the efficient selective “human host depletion” NAS sequencing and species-specific ARGs prediction. The microbial sequence yield was increased by a factor of at least 8-fold in 21 sequenced clinical bronchoalveolar lavage fluid samples, and the process was completed within 4.5 h from sample to result (57). This ultra-sensitive diagnosis of bacterial pathogens and ARGs from clinical samples has the potential to reduce unnecessary prophylactic antibiotic use and the related morbidity and mortality.

### 4.3 Limitations

To our knowledge, this study is the first bibliometric analysis to comprehensively describe the latest developments in nanopore sequencing in the field of pathogenic microorganisms. It elucidates early collaboration patterns, journal contributions, and research hotspots in this field, aiding scholars in identifying knowledge gaps and future directions. However, this study has some limitations. First, our analysis relied solely on the WOSCC, a constraint inherent to bibliometric methods. We mitigated database bias through rigorous search strategies and manual verification to ensure that the results obtained in this study are a true reflection of the existing literature. Second, while bibliometrics quantifies macro-level trends, it may overlook nuanced insights within the individual studies. Additionally, nanopore sequencing faces challenges, including lower raw read accuracy (96.84% for nanopore versus 99.68% for Illumina), high costs, and underdeveloped bioinformatics pipelines (58).

## 5 Conclusion

In summary, the rapid growth of nanopore sequencing research from 2014 to 2024 highlights its potential for pathogen diagnostics. While current limitations exist, we believe that they are not insurmountable technical obstacles but catalysts that promote continuous innovation. By integrating bioengineering, artificial intelligence, and public health strategies, this technology may achieve breakthroughs in ultrasensitive detection (detection of pathogens with a load as low as 1 copy/μL within 1 h), fully automated analysis (handheld devices for sample-to-clinical report), and global genomic surveillance networks, ultimately transforming infectious disease management.

## Data Availability

The original contributions presented in the study are included in the article/Supplementary material. Further inquiries can be directed to the corresponding authors.

## Glossary

EBOV: Ebola virus
ZIKV: Zika virus
SARS-CoV-2: Severe Acute Respiratory Syndrome Coronavirus 2
WOSCC: Web of Science Core Collection
*E. coli*: *Escherichia coli*
HPV: Human papillomavirus
*M. tuberculosis*: *Mycobacterium tuberculosis*
HSV: Herpes simplex virus
DENV: Dengue virus
HCV: Hepatitis C virus
IAV: Influenza A virus
MPXV: Monkeypox virus
*S. aureus*: *Staphylococcus aureus*
*K. pneumoniae*: *Klebsiella pneumoniae*
*S. pneumoniae*: *Streptococcus pneumoniae*
*P. aeruginosa*: *Pseudomonas aeruginosa*
CHIKV: Chikungunya virus
HAdV: Human adenovirus
AMR: Antimicrobial resistance;
ONT: Oxford Nanopore Technology;
NTS: Nanopore Targeted Sequencing
HCPs: Highly Cited Papers
*A. baumannii*: *Acinetobacter baumannii*
LRI: Lower respiratory infection
RSV: Respiratory syncytial virus
NAS: Nanopore adaptive sampling
