# Correction: Global trends in the application of nanopore sequencing technology in the detection of infectious disease pathogens: a bibliometric analysis from 2014 to 2024

**DOI:** 10.3389/fmed.2025.1678005

**Published:** 2025-09-05

**Authors:** Jiali Long, Benhua Zeng, Jia Li, Juan Zhang, Guohong Deng

**Affiliations:** ^1^Department of Infectious Diseases, Southwest Hospital, Third Military Medical University (Army Medical University), Chongqing, China; ^2^Chongqing Key Laboratory of Viral Infectious Diseases, Chongqing, China

**Keywords:** nanopore sequencing, pathogenic microorganisms, bibliometric analysis, real-time, genomic surveillance, antimicrobial resistance

Upon review, we noticed that in Figure 3B, the Taiwan region of China should have been colored red to indicate >101 publications, consistent with our statistical analysis. This was inadvertently omitted during the figure generation using the rnaturalearth R packages. The corrected Figure 3 and its caption appear below.

**Figure 3:**
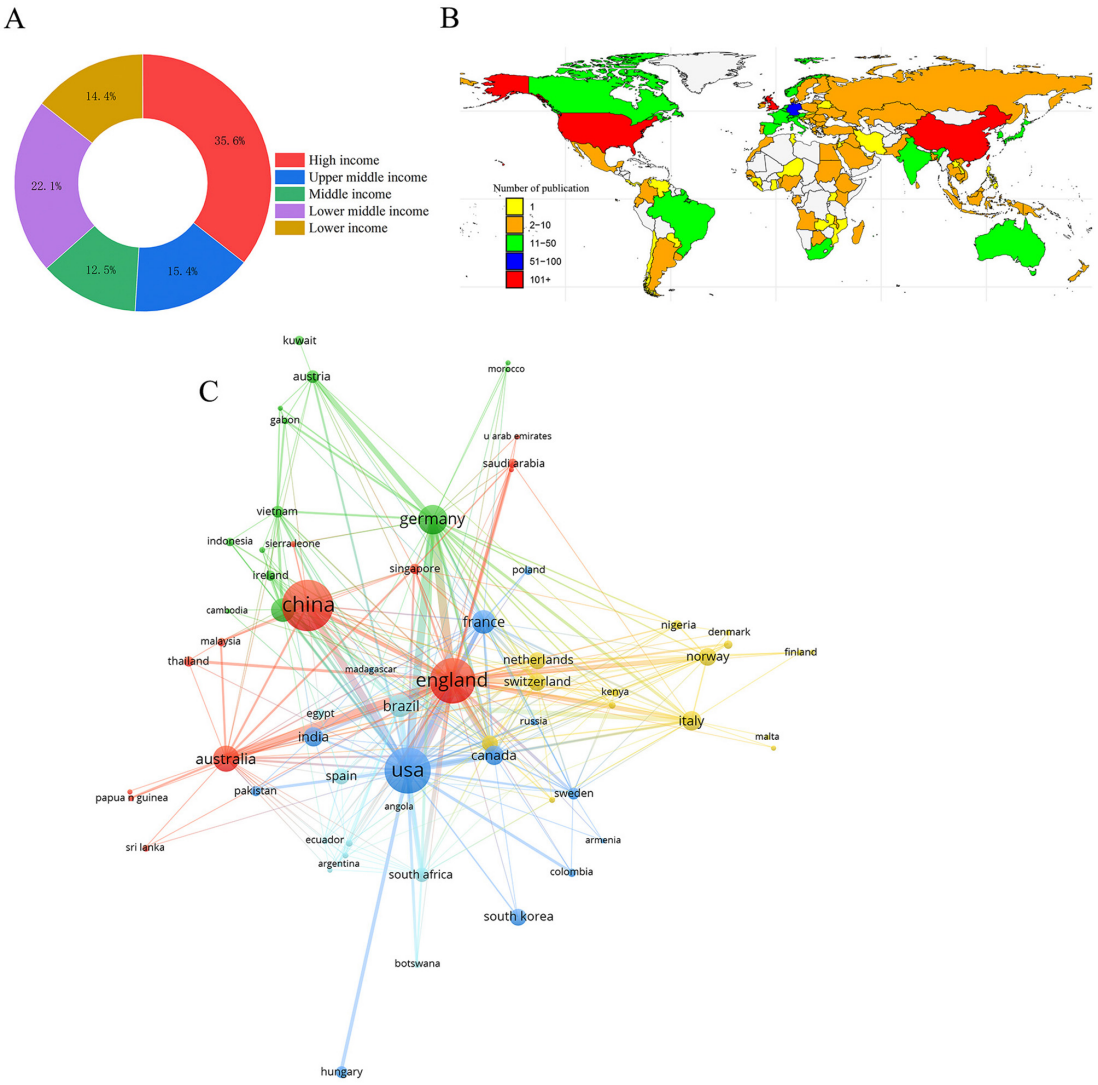
Relationships and clusters of countries. **(A)** Classification of countries according to income levels. **(B)** Geographical distribution based on the number of documents. **(C)** Relationship between international and domestic collaborations.

The original article has been updated.

